# Colloidal Force Study of Particle Fouling on Gas Capture Membrane

**DOI:** 10.1038/s41598-017-13553-3

**Published:** 2017-10-11

**Authors:** Lin Zhang, Bin Hu, Hang Song, Linjun Yang, Long Ba

**Affiliations:** 1 Key Laboratory of Energy Thermal Conversion and Control, Ministry of Education, School of Energy and Environment, Southeast University, Nanjing, 210096 China; 2 State Key Laboratory of Bioelectronics, School of Biology and Medical Engineering, Southeast University, Nanjing, 210096 China; 3 Key Laboratory of Coal-Based CO2 Capture and Geological Storage of Jiangsu Province, China University of Mining and Technology, Xuzhou, Jiangsu 221008 China

## Abstract

Membrane fouling induced by industrial flue gas deteriorates their gas capturing efficiency, which is mainly caused by the adhesion of aerosol particles. To fully understand the mechanism of membrane fouling, a quantitative study of the adhesion force of particle on membrane surface was investigated by atomic force microscopy (AFM). The adhesion force of a single particle with flat glass, silicon wafer, PP (polypropylene) membrane, and fly-ash particles were measured within the relative humidity (RH) of 0 ~ 85%. The results showed the adhesion force of a particle with membrane have not much difference from the glass and silica wafer. And the surface roughness of flat substrate has slight effect on the adhesion force of the micrometer scale particle on flat surface at dry condition, while measured adhesion forces show obvious RH dependent for glass and membrane. Additionally, at dry conditions, the adhesion force of inter-particles also shows no obvious quantitative difference but obvious scattering comparing to that on membrane. The adhesion force of inter-particles increased more higher with the RH than that on membrane, which indicates the adhesion between micrometer scale particles can accelerate the deposition of particles on membrane and contributes the most to membrane fouling in industry atmosphere.

## Introduction

Coal-fired power plants contribute at least 40% of CO_2_ emissions^1^, which makes the CO_2_ emission control for the power plants crucial important^2,3^. Encountering to the challenge of climate change, the technologies of the emission reduction, capture and utilization of greenhouse gas are exhibiting ever-increasing imminent importance^4–6^. Membrane separation/adsorption technology is an option for post-combustion CO_2_ capture. Compared to the traditional skills, such as amine scrubbing and sorbent adsorption, membrane separation shows better prospect for industrial application due to extensibility and modularity, cost efficiency and lower energy requirement^7–11^. The polymer membranes such as PP, PVDF, PTFE (Polytetrafluoroethylene), PSF (polysulfone) are selected for CO_2_ separation and capture due to the high chemical and thermal stability^12–14^. However, the membrane is vulnerable to pollutions like aerosol particles^15–17^. Colloids could deposit and attach on membrane to form cake layer resulting in the decrease of membrane performance and destroy of membrane structure^18,19^. Thus particles induced membrane fouling has become the chief obstacle for the industrial applications of membrane, which arise increasing attention.

Generally, the fine particles in industry gas are micron or submicron particles sphere^20^. These micro-particles are difficult to be observed and detected by using conventional means. Atomic force microscope (AFM) was originally developed by Ducker *et al*.^21^ and provides an efficient method to study the microcosmic contact interaction between particles and membrane. Numerous investigations studied the interaction of pollutants and membrane surface by using AFM and analysis the mechanism of membrane fouling^18–31^. In most previous investigations, the fouling of membranes by foulants in water treatment was studied by using silica and polystyrene colloids through AFM. Bowen *et al*.^22–24^ studied the adhesive force between a colloid probe and polymeric ultrafiltration membranes (ES 404 and XP 117) by using AFM as well as the effect of the surface roughness on particle adhesion. Boussu *et al*.^25^ suggested that the membrane hydrophobicity and roughness seems to play a more significant role for promoting fouling. Lee and Elimelech *et al*.^26–28^ use AFM to determine the adhesion force between bulk organic foulants and foulants deposited on the membrane surface to study organic fouling of reverse osmosis membranes. The results indicated that the rate of initial membrane fouling and the membrane cleaning efficiency has relationship with the colloid-membrane interaction force. For industry gas treatment, Bram *et al*.^18^ and Brands *et al*.^19^ found the separation performance of Co-SiO_2_ membrane, ceramic membrane (Ti_0.5_Zr_0.5_O_2_) and polymer membrane decreased dramatically after long-term exposure in flue gas. And the surface of membrane was covered by fine particles totally and the microstructure was damaged significantly. In our previous lab work, it was also found that the fine particles in flue gas obviously decreased the performance and damaged the mirco-structure of membrane during CO_2_ capture process^29–31^. Particle induced membrane fouling is closely related to the adhesion force of aerogel. Other studies have shown surface roughness, RH, particle size affect the adhesion force of aerogel to different extent in air circumstance^32–35^, however the existing results shown there are obvious variations between different materials. Therefore, quantitative study of the adhesion force of particle on the gas CO_2_ capture membrane surface is necessary, which helps to better understand the mechanism of colloid induced membrane fouling in process of gas separation.

This work is motivated by the need to study the fouling of fine particles on membrane during the CO_2_ capture from industry flue gas and to quantitative study of the adhesion force of particle on the membrane surface. The particle probe was prepared by attaching a SiO_2_ sphere as a fly-ash particle to the tip of atomic force microscopy (AFM) cantilever to study the adhesion force of particle with different substrates. The adhesion force of SiO_2_ particle with glass, silica wafer, PP and PDVF membrane was measured by using AFM under the relative humidity (RH) range of 0~85%. The surface roughness and morphology of membrane were characterized by AFM. The effects of surface roughness, RH and surface physic-chemical property were investigated. Furthermore, the inter-particles adhesion was investigated by contacting SiO_2_ sphere to fly-ash particles to reveal the inter-particle contact behavior and its relationship to bulk cohesion.

## Results and Discussion

### Fouling of Fine Particles on PP Membrane During CO_2_ Capture From Flue Gas

**Figure 1:**
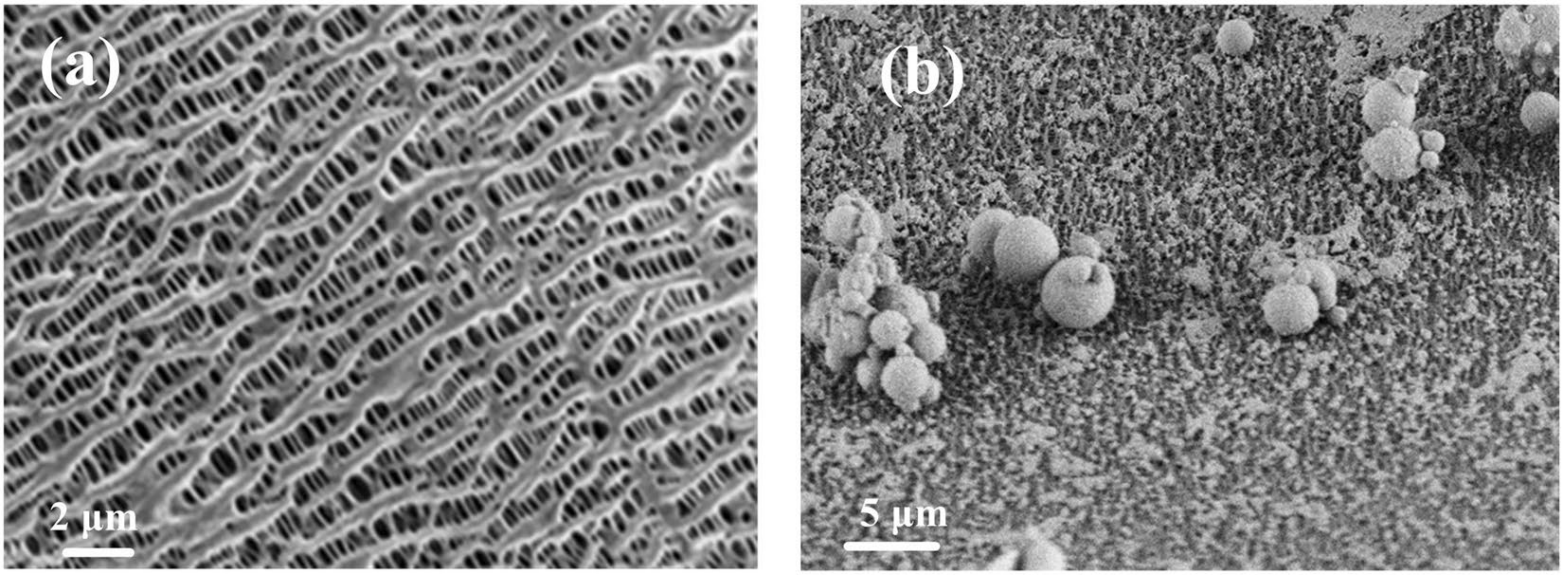
FESEM images of PP hollow fiber membrane (**a**) fresh membrane (**b**) membrane fouling by fine particles.

In our previous study^30^, the membrane absorption for CO_2_ capture by using PP hollow fiber membrane was conducted under the actual coal-fired flue gas conditions for 7 h, resulting in the capture properties decreased about 20%. The morphologies of the fresh and used membrane were studied here by SEM and shown in Fig. 1. As shown in Fig. 1(a), the pore structure of the fresh membrane is clear, and the pore size is ca 0.1~0.5μm. However, a severe membrane fouling was observed over used membrane. Shown in Fig. 1(b), the surface of membrane was covered by a large amount of fine particles. Some of these fine particles adhered on the surface of membrane individually or in clusters. At top left corner in Fig. 1(b), a cake-layer like particles was observed on the surface of membrane, which may be piled up by some extreme small and unrecognizable particles. The recognizable particles in the picture are spherical and are identified as coal-fired fly-ash. The diameter of those observed particle is approximately in range of 1~10 μm. Generally, the mainly components of fly-ash is SiO_2_^36^, which is confirmed by the results of EDS, shown is Table 1. Therefore, in order to study the fouling of membrane by fly-ash, the SiO_2_ microsphere with diameter of 11.6 μm was selected to prepare fine particle colloid probes to study the adhesion force of fly-ash particle on membrane over AFM.

**Table 1:** The EDS of the particles deposited on membrane.

Element	C	O	Mg	Al	Si	K
Weight percent (%)	43.20	18.93	0.21	1.05	28.34	3.46
Atom percent (%)	59.56	19.59	0.14	0.65	16.71	1.47

### Surface Characterization of the Substrate

**Figure 2:**
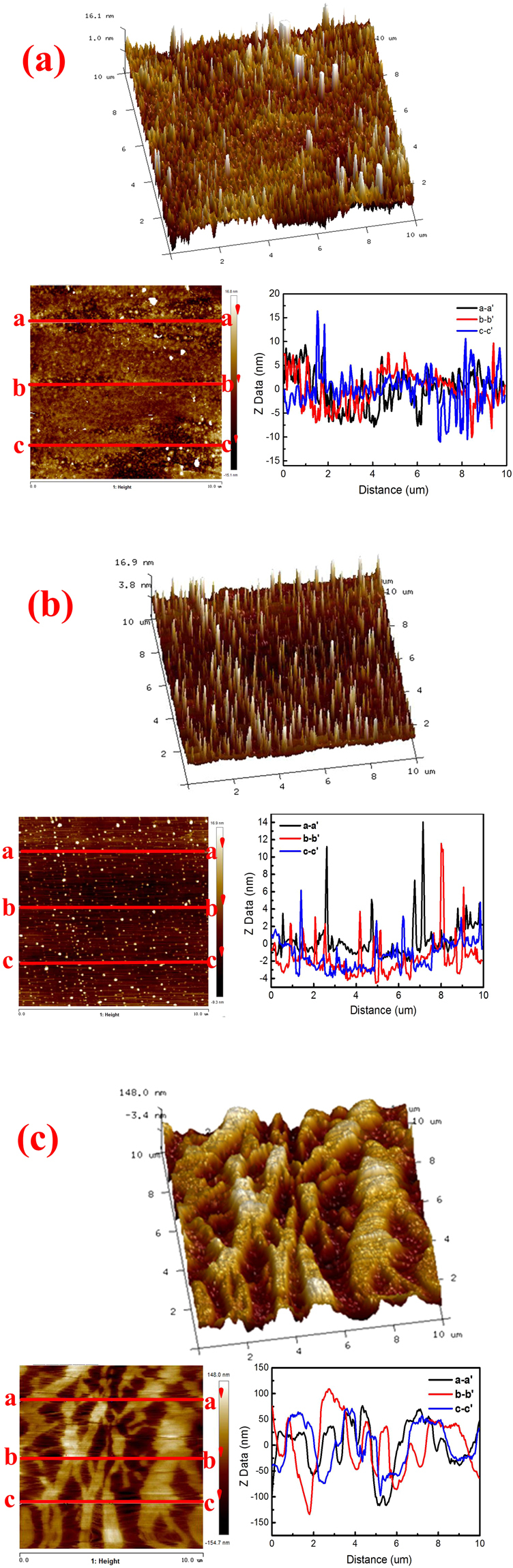
AFM topographic images of (**a**) glass (**b**) silica wafer (**c**) PP membrane.

Prior to collecting the adhesion force between particle and substrates, the surface morphology of glass, silica wafer and PP membrane were characteristic using AFM and the 3D and height images are presented in Fig. 2. The surface roughness of the four substrates was analyzed through random three sections. The RMS roughness and the Ra roughness of all surfaces were listed in Table 2. The glass (Fig. 2(a)) has a regular and uniform morphology with relative small roughness (RMS = 4.37 nm, Ra = 2.86 nm). Silica wafer (Fig. 2(b)) shows regular and super smooth surface with 1.35 nm RMS roughness and 1.36 nm Ra roughness, which has the smoothest surface among the four materials. As comparison, the surface PP membrane is rather asperity. The roughness of PP membrane is RMS of 54.8 nm. The surface PP membrane has much higher roughness than glass and silica wafer. Furthermore, the surface of PP membrane shows special streaky folds, reflecting its miro-pore structure shown in Fig. 1(a). Moreover, as the 3-D images and the roughness of the three sections shown, the surface morphology of surfaces of glass and silica wafer substrate are similar in all directions, which could be regarded as similar isotropy. The surfaces of PP membrane are not uniform, the morphology and roughness of them are different in each direction, which could be identified as anisotropy surfaces^37^.

**Table 2:** The roughness of the substrate.

Sample	Glass	Silica wafer	PP membrane
RMS roughness Rq (nm)	4.37	1.35	54.8
Average roughness Ra (nm)	2.86	1.36	51.1

### Adhesion Force of Particle to Substrate: effect of Roughness

**Figure 3:**
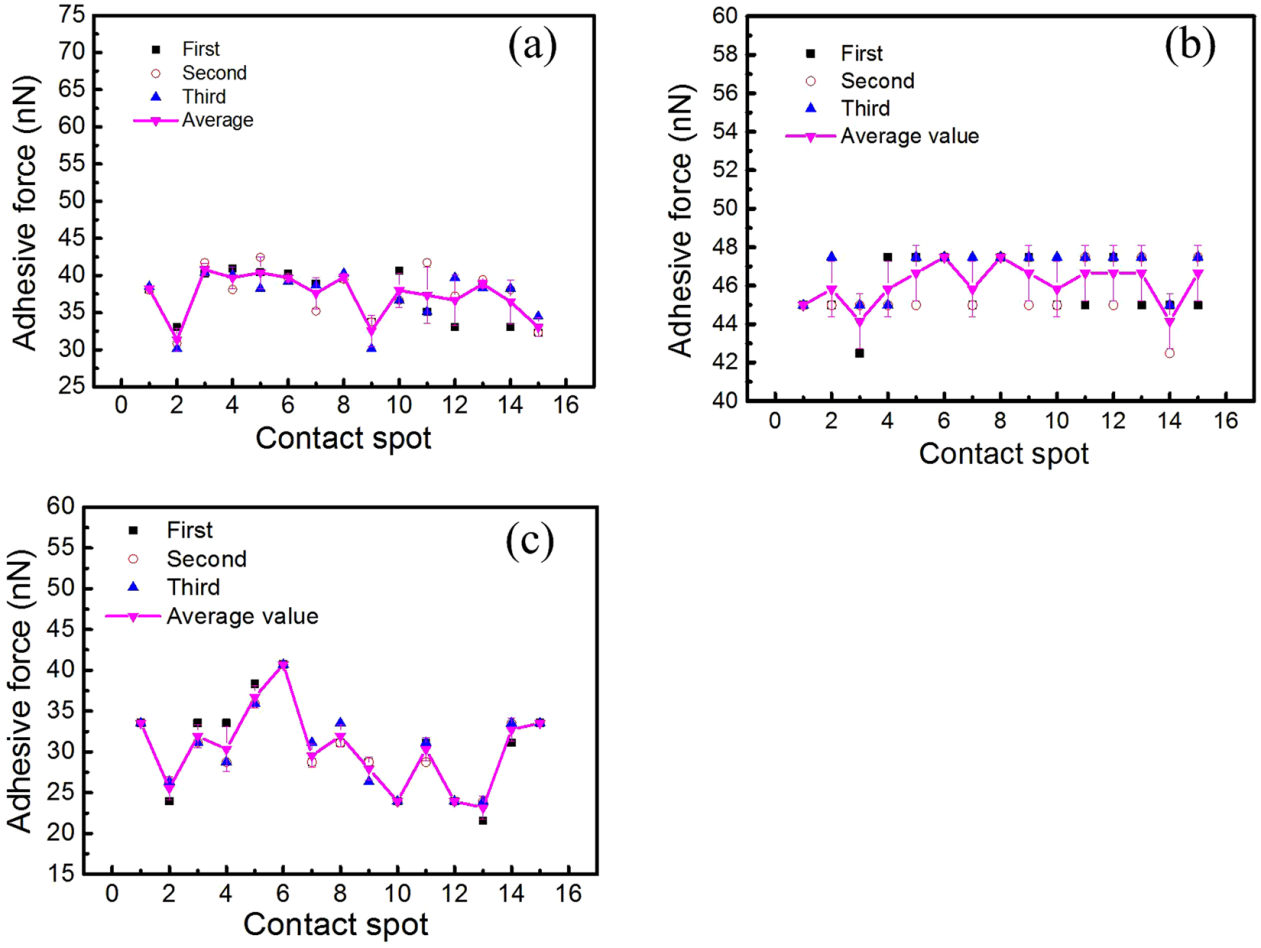
Adhesion force of between the SiO_2_ particle with (**a**) glass (**b**) silica wafer (**c**) PP membrane at RH = 0~5%.

The adhesion force between fine particles and the substrate was measured in the liquid cell of the AFM at dry circumstance (RH = 0%) to eliminate effect of water vapor in air. Fifteen contact spots were selected to measure the adhesion force of fly-ash particle on each substrate of materials. And three pairs of force curve (entrant and retract cycles) were collected at each spot to calculate the adhesion force. The adhesion force of fly-ash particle on glass, silica wafer and PP membrane is shown in Fig. 3. For each contact spot, the adhesion force obtained by three times of force curves are basically consistent, showing the good repeatability. For the glass and the silica wafer substrate, the adhesion forces are not only consistent in the same spot but have small difference from each contact spot to another. In another word, the adhesion force is less of dispersive for the glass and silica wafer in this experiment. However, it is different for PP membrane. As shown in Fig. 3(c), the adhesion force of particle with PP membrane has larger difference between each contact spot and has larger dispersity. This may be explained by the surface character of the materials. As discussed above, the surface of glass and silica wafer is similar isotropous and the roughness of each contact spot is very small, therefore, for a micro scaled particle, this contact is similar to the idealized adhesion of sphere - flat surface. The contact area is approximate to constant. Consequently, the adhesion force obtained at each spot exhibit small difference. While, for PP membrane substrate, the surface is much more irregular and the roughness is more different from one spot to another due to the anisotropy. Therefore, the contact area and interaction behavior of the same fly-ash with different contact spot on PP membrane are of large different. Consequently, the adhesion force exhibit greater dispersity.

Because the exact value of the adhesion force is vary between contact spot due to the difference of the contact area and the geometry. In order to better compare the adhesion force of fly-ash with the four surface substrates, the adhesion force obtained at fifteen contact spot were input to statistical analysis. The statistic values of the adhesion force were listed in Table 3. The rank of the adhesion force between a SiO_2_ particle and the four material substrates could be obtained in the order silica wafer > glass > PP membrane. This may be also caused by the surface roughness. The roughness of glass (RMS = 4.37 nm) and the silica wafer (RMS = 1.35 nm) are much lower than that of PP membrane (RMS = 54.8 nm). The contact area is larger for a smooth surface with little roughness than that of a surface with high roughness, contributing larger adhesion force^38^. Moreover, the rougher surface also results in the wider distribution of adhesion force. However, the difference of the adhesion force between the four substrates is not obvious. The adhesion force of a SiO_2_ sphere with membrane has not much difference from the glass and silica wafer, which is about several dozens of nN. This implies the surface owing nano-sized roughness has slight effect on the adhesion force against surface for a micron-diameter fly-ash particle.

**Table 3:** The statistic values of the adhesion force of particle on the substrates.

Parameters	Adhesion force		
	Glass	Silica wafer	PP membrane
Average values (nN)	37.9	46.1	30.4
Standard deviation (SD)	2.93	1.02	4.94
Minimum values (nN)	31.3	44.2	23.2
Maximum values (nN)	40.8	47.5	40.7

Here, the contact in dry and completely free from capillary force effects can probably be explained by JKR theory. The surface free energy for clean glass or silicon approximate is 35 mJ/m^2^ and for PP is 26 mJ/m^2^. The calculated value for glass, silica wafer and PP membrane are 3826.5 nN and 3298.9 nN, which are much larger than the experiment values (46.1 nN and 30.4 nN). This discrepancy might due to that the surface energy calculating used is too large and the uncertainties surface geometry, which is neglected in the JKR equation.

### Effect of Relative Humidity

**Figure 4:**
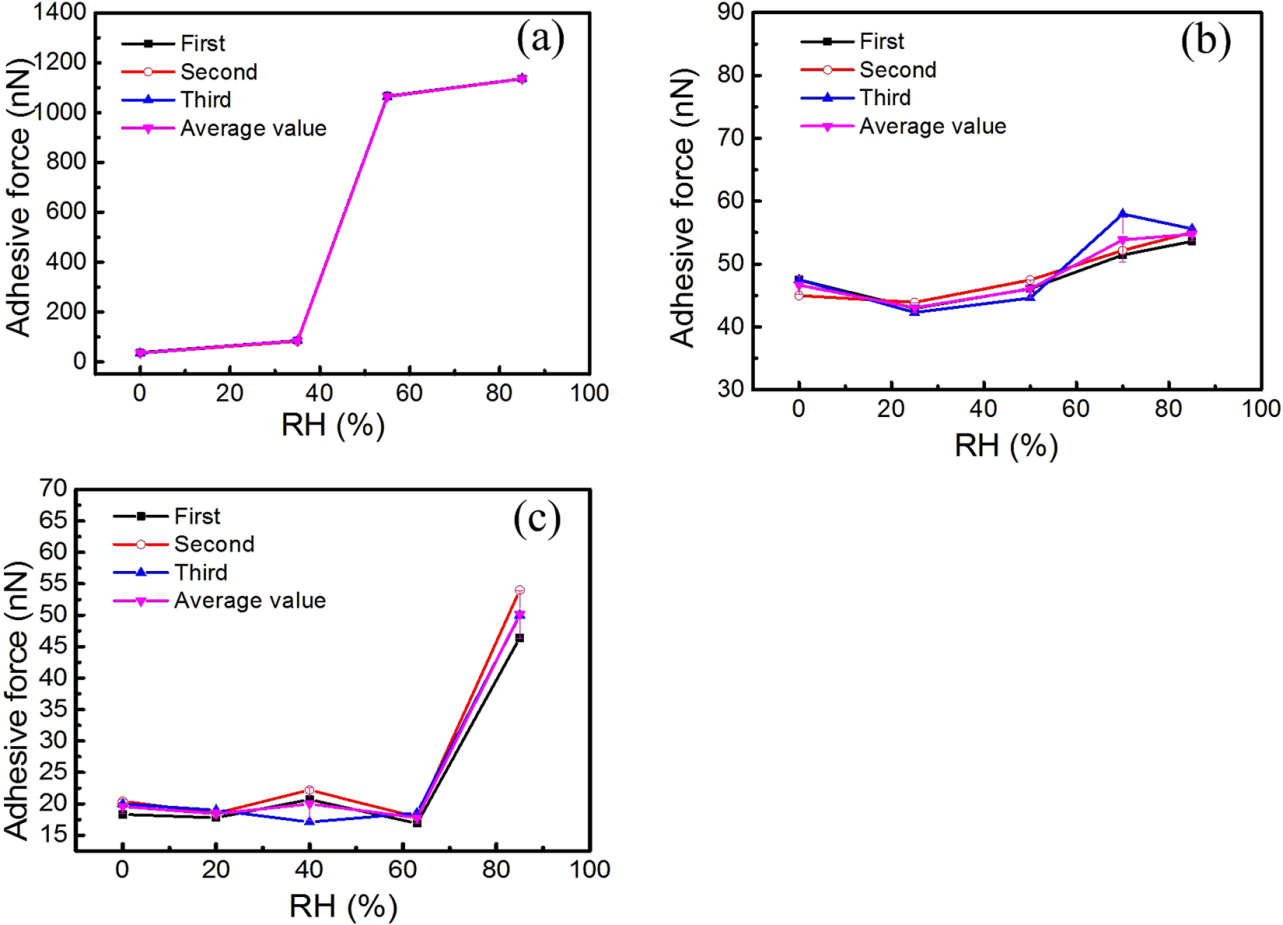
The RH dependence of adhesion force of the fly-ash particle with (**a**) glass (**b**) silica wafer (**c**) PP membrane.

In the coal-fired power plant, the CO_2_ membrane capture system are suitable to installed following the WFGD system and the flue gas usually contains 5~10 wt% of water vapor^39^, which could influence the adhesion behavior of the fly-ash particles. Therefore, the adhesion force of fly-ash particle on the four material substrates was measured at the RH range of 0~85%. The dependence of the adhesion force on RH was shown in Fig. 4. As shown in Fig. 4(a), the adhesion force of the fly-ash particle with the glass dramatically increased with the increase RH, which could be attributed to capillary force. Both the glass substrate and the fly-ash particle represent a hydrophilic surface, which exhibits strong affinity to water vapor and tend to form a water film on the surface^40^. The water film will generate a large capillary force for the fly-ash particle, therefore, contributes the large adhesion force. In contrast, no obvious increase is observed for the adhesion force of fly-ash particle with silica wafer even there is a little decrease when the RH increased from 0 to 25%. The possible reason is the surface of silica wafer is hydrophobic, which is hard to attract vapor to form the liquid bridge^41,42^. Therefore, the capillary force due to the high RH makes little contribute to the total adhesion force. Moreover, the small decrease at the RH range of 0 to 25% may due to the release of the electrostatic force. Generally, surface chemistry, geometry, electrostatic force, and capillary forces affect the adhesion force of a particle to surface^43^. The electrostatic force is much stronger in dry circumstance than in wet circumstance^44^. Hence, the adhesion force of the fly-ash particle to the silica wafer exhibits small decrease when the RH increased from 0 to 25%.

**Figure 5:**
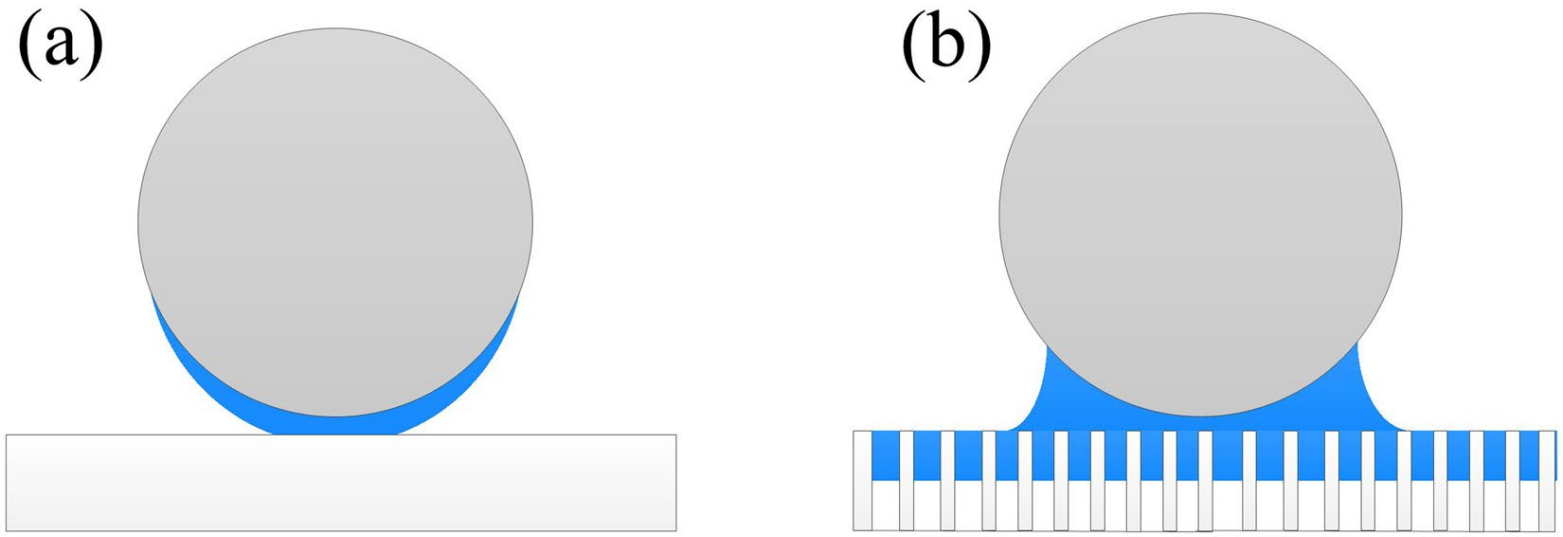
Schematic of the interacting of particle on (**a**) silica wafer (**b**) PP membrane at high RH.

As shown in Fig. 4(c), the adhesion force of the fly-ash particle to PP membrane is almost constant at the RH range of 0~60%, however, exhibits increase with the RH approaching to 85%. The result could be ascribed to the combined effect of hydrophobicity, the surface micro-structure and the roughness of PP membrane. On one hand, PP membrane is hydrophobic surface, the water film is hard to form between hydrophobic surfaces. Hence, the RH shows no obvious effect on the adhesion force of the fly-ash particle to PP membrane at low RH. The van der waals force, contact force and week electrostatic force contribute the main body of the adhesion force, which has no dependence on the RH. On the other, the PP membrane has the micro-pore structure surface as shown in Fig. 2(c), and Fig. 1, small pore is favorable to the capillary condensation of water vapor^45,46^ especially at high RH. In this case when the particle probe contact with the surface, the particle may contact with water and also generate additional liquid bridge force, although no successive water film formed, as shown in Fig. 5. Furthermore, the fly-ash particle is a hydrophilic surface, which may be wetted at high RH (Fig. 5(b)). Therefore, the capillary force still makes a contribution to the adhesion force at high RH.

### The Contact Force of Inter-particles

**Figure 6:**
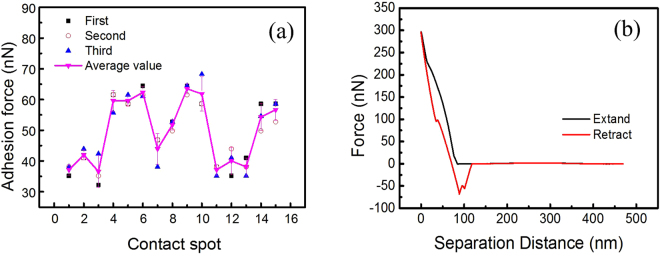
Adhesion force of between the SiO_2_ and fly-ash particle at RH = 0~5% (**a**) adhesion force (**b**) typical force curve.

As shown in Fig. 4(b), the fly-ash particles not only adhered on the surface of the membrane but some of them agglomerated together or adhere on other particles. In order to understand the interaction behavior between the particles, the inter-particle adhesion force was studied in this section. The fine-particle probe with spring constant is 2.56 N/m was used to collect the force curve through contacting a glass surface fixed by a layer of fly-ash particles. The adhesion force of the random fifteen contact spot and their statistic values are shown in Fig. 6 and Table 4. As seen from Fig. 6(a), the adhesion force between fly-ash particles mostly exhibits consistent at the same contact spot except for spot number 10, where the maximum value (68.26 nN) is obtained. This may be caused by the multi-point contact while collecting the force curve, which is observed through the force curve shown as Fig. 6(b). This multi-point contact could be ascribed to two folds. First, unlike the flat surface, the surface of fly-ash particle is spherical with a certain rate of curvature and some fine particles may attached on the bigger ones. Therefore, when the particle probe approach vertically to the fly-ash the particle probe may firstly contact with the top of the bigger particle, then slide down to contact the smaller one. Moreover, the particle probe may contact two or more particles at the same approaching.

**Table 4:** The statistic values of the adhesion force between particles.

particle-particle Adhesion force	Minimum value (nN)	Maximum value (nN)	Average value (nN)	Standard deviation (SD)
	35.16	68.26	49.63	10.6

As the statistic data shown in Table 4, the minimum value and the average adhesion force of the particle with the fly-ash particle is 35.16, and 49.63 nN, respectively, which is similar to the adhesion force of particle with Silica wafer and a little higher than those of PP membrane. This result implies the surface characteristic of the contact area has slight influence on the adhesion force under dry condition in this work. However, the standard deviation of adhesion force data obtained through the contact of particle-particle is 10.6, which is much higher than the case with the flat substrates (Table 3). This indicates the distribution of adhesion force between fly-ash particles is much higher compared to the flat surface. This could also attribute to the complicated surface contact between particles.

**Figure 7:**
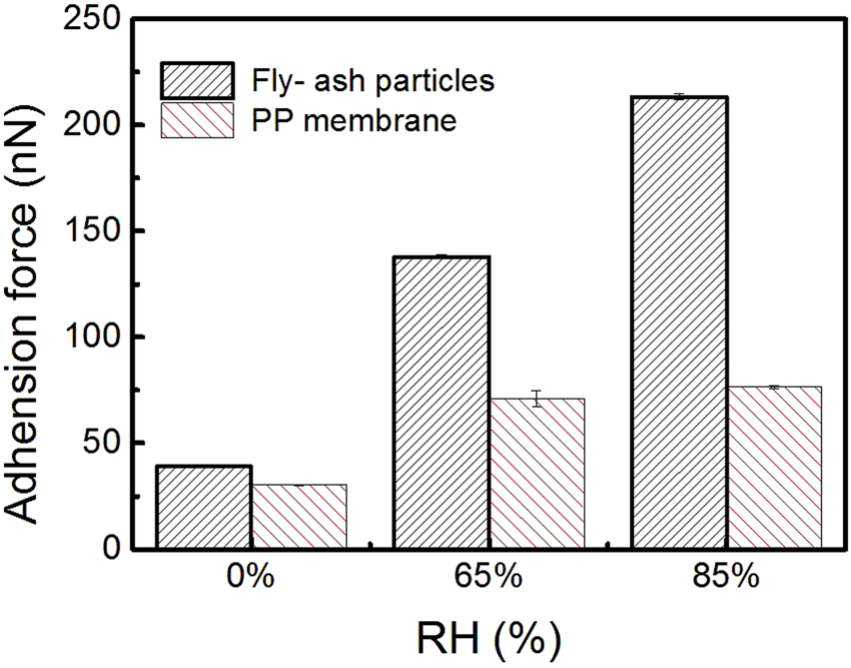
Adhesion force of between the SiO_2_ and used PP hollow fiber membrane at different RH.

In order to simulating the actual conditions of power plant, the effect of RH on the adhesion force between particles was studied. Generally, the humidity could affect the deposition state of fly-ash particles and the formation of cake-layer^18,29–31^ because RH mainly influences the moisture content and the adhesivity of fly-ash. When RH increased, the moisture content and the adhesivity of the fly-ash increased due to the adsorption of water molecule, which cause fly-ash -particles tend to adhered to each other^47,48^. However, the fly-ash particles were fixed on the glass surface before collecting the inter-particle adhesion force. Therefore, adjusting the RH, the state of the fly-ash will not change basically. Shown in Fig. 7, as the RH increased from 0s to 85%, the adhesion force of particle with fly-ash particle increased from 40 nN to 213 nN. This significant increase could mainly attribute to the colloid force due to the liquid bridge, which formed gradually as the increase RH. The adhesion force increased by 448%, which is much higher than the increase percentage of the adhesion force of particles on PP membrane with 151%. This result could be ascribed to the geometrical morphology and the physic-chemical properties of fly-ash particles. Generally, the colloid force between particles includes two parts: (a) the surface tension of the liquid film formed between solid and liquid, (b) hydrodynamic force due to low capillary pressure formed by average of curvature of liquid surface^49,50^. On one hand, the fly-ash contains plenty of water soluble ions (ie, Cl^−^, K^−^), which could dissolve into the liquid film when RH increase. Those ions tend to attract the H_2_O molecule into the liquid bulk, results in the increase of the surface tension^51^. On the other, the hydrodynamic force of inter-particles is higher, due to the pendular liquid bridge over sphere-on-sphere^52,53^. This special pendular bridge geometry gives additional surface tension component of adhesion^54^, which is previously ignored in Eq. (2). The above results implies the adhesion force of inter-particles is much stronger than the adhesion force of particle with membrane at high RH, which results in significant deposition of fly-ash particles on membrane and play a vital role in membrane fouling.

In conclusion the contact interaction of a single particle with typical PP membranes and the inter-particles were studied through the SiO_2_ particle probes by using AFM. By comparison of the glass, silica wafer, PP membrane, the results indicate that the surface roughness and nanoasperity contacts has slight effect on the adhesion force of the micrometer scale particle for the flat surface at dry condition. Moreover, although the membrane and silica wafer are hydrophobic materials, the adhesion force of particle with membrane showed obvious RH dependence due to the special micropore structure, which is opposite to the case of silica wafer. Additionally, at dry conditions, the adhesion force between colloid and particles also shows no obvious quantitative difference but obvious scattering comparing to that on flat substrate, which was explained by the multi-point contact between particles. The adhesion force of the SiO_2_ colloid on the particles increased more obvious with the RH than that on membranes, which indicates that the adhesion between micrometer scale particles can accelerate the deposition of particles on polymer surface and attributes the most to the membrane fouling within industry atmosphere.

## Methods

### Materials

SiO_2_ microsphere (Sil-N-11015) is selected to simulate the fly-ash particle, which is purchased from Sphere Scientific Co., Ltd., China, in size of approximate diameter 11.6 μm. The cantilevers of spring constant 0.61 N/m and 2.56 N/m (contact silicon cantilever, CSC 12/50, Ultrasharp) were used to prepare fine particle probes and measure the adhesion force. The propene polymer (PP) hollow fiber membrane supplied by Hangzhou Kaihong Membrane technology co., Ltd., China respectively was used without further treatment. The glass and silica wafer material were also used as the substrate and contrast to PP membrane. The glass sheet pretreated by acid and the silica wafer sheet were ultrasonic rinsed and dried before measurement.

### Characterization

The morphology of membranes and the micrograph fine particle modified probe was observed using field emission scanning electron microscopy (FESEM, Model FEI SIRION 200). The elementary composition of the fine particles adhered on the membrane was detected by Energy Dispersive X-ray Detector (EDS). The rendered 3D surface morphology were studied by using Multimode-8-AFM (USA/Banker Nano Inc.) in standard tapping mode in air and the scan size is 10 × 10 μm. And the surface roughness of all the substrates was analyzed by Nanoscope analysis software. The root-mean-square roughness (RMS) and average roughness (Ra) were selected to describe the surface roughness of the material. The SiO_2_ spheres were much less rough than the most substrates and were regarded as smooth surface.

### Particle colloidal probe preparation

**Figure 8:**
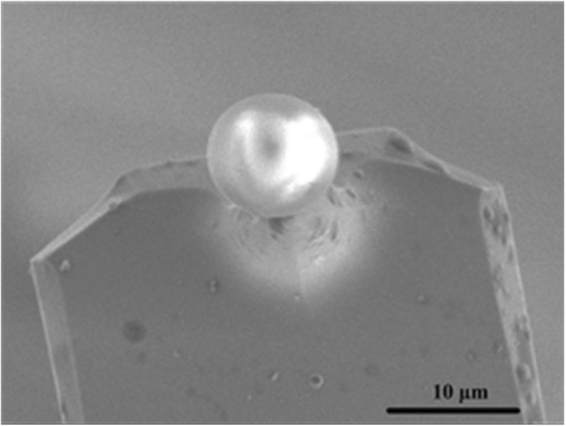
SEM image of a SiO_2_ sphere modified AFM cantilever.

The colloid probes were prepared firstly. The SiO_2_ spheres were attached to the cantilevers with epoxy resin by using a micromanipulator under the observation of the high magnification optical microscope or the high power video detection. The cantilever was firstly dipped into appropriate amount of epoxy resin by using the AFM controls and then was moved to attach a single particle through adjusting the sample stage and the piezoelectric loading. The typical SEM image of the fine particle modified probe is presented in Fig. 8.

### Adhesion Force Measurement

**Figure 9:**
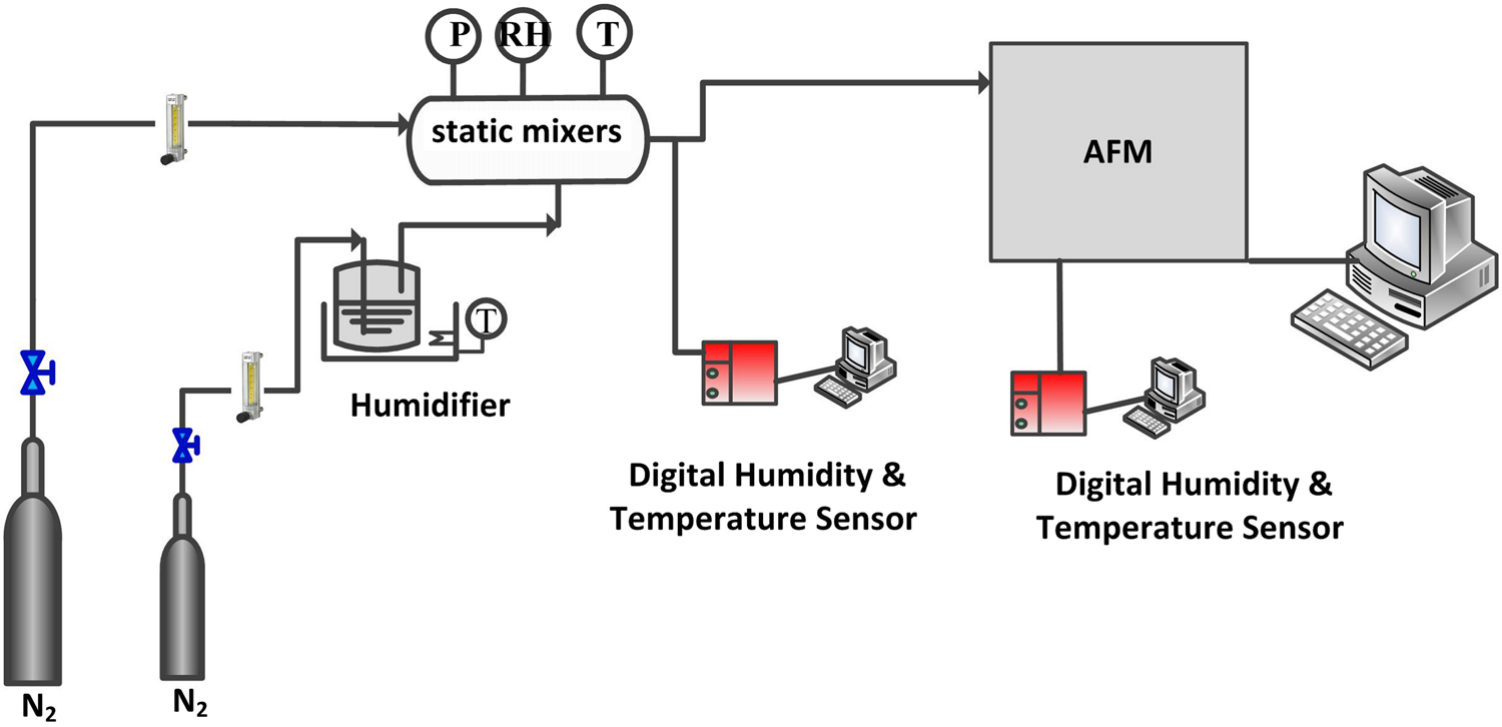
Moisture control and adhesion force measurement system.

The adhesion force measurement was conducted over a RH adjustable-AFM system shown in Fig. 9. The force-cured collected over a liquid cell of AFM. The RH of the measurement circumstance was controlled through the gas continuously flow, which contains a dry line of nitrogen and a humidifying line containing a tank of water. The RH was detected by the HMT337 humidity transmitter covering the range 0~85%. The AFM operation and the force measurement were not affected by the air flow. The RH in the liquid cell could reached the desired value within 10 min by continuous gas flowing and keep stable. For study the adhesion force at dry conditions, the adhesion forces were collected at fifteen randomly selected contact spots on each substrate surfaces. And three pairs of force (entrant and retract cycles) were collected at each spot. While investigating the effect of humidity, the force curves were collected three times at one point before changing the RH. The operation parameters used were as follows: the set point 0.5 V; scan size is 1.0 nm; Scan rate is 1.0 HZ.

**Figure 10:**
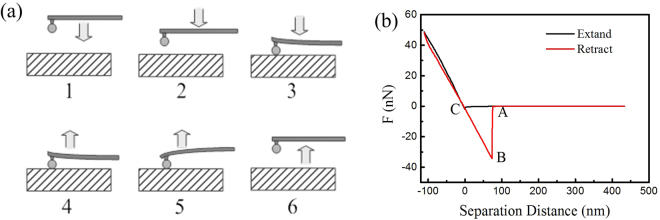
Schematic diagram of adhesion force measurement process (**a**) extant and retract (**b**) force curve measured by the fine particle colloidal probe.

The principle of force measurement is described as Fig. 10(a). The tip of the cantilever is attached a fine particle, when the probe approach to the surface, the cantilever appeared distorted due to the coefficient of attraction and repulsion and produced a signal proportional to the deflection of the cantilever as a function of the displacement of sample^55^. The adhesion force was obtained through converting the deflection data into the force curve as the function of the displacement of sample. The Fig. 10(a) 1–3 and 4–6 presents the force situation during the approaching and retracting of the probe, corresponding to the force curve A-C-B-A in Fig. 10(b). At the beginning, the separation distance is large which is out of the affecting range of van der waal’s force hence the cantilever did not bend, as the probe approaching to the surface continuously, the probe jump to the surface s due to the attraction, which reflects as point C in Fig. 10(b). The cantilevers continue bend until the loading reach the set-point, where after the cantilever retracted and the sample displacement became larger. However, the probe was not separated immediately from the substrate due to the attraction but reversed bending as describe in Fig. 10(a) 4–5, which reflects as point A to point B in force curve and reached the maximum at point B. Finally, the probe jumped off the surface as the sample separation distance becoming larger and the force curve collection was accomplished. The maximum of force to separate is the regarded as the maximum adhesion force.

### Theoretical Background

The interaction forces of fine particles with a surface mainly include van der waals forces, contact force, capillary forces and electrostatic forces. For hydrophobic surfaces at low RH, the idealized adhesion force of sphere - flat surface is determined to be described by Johnson-Kendal-Roberts (JKR) theory^56^. The JKR theory is applicable for the cases with highly deformable contacts or the surface with high surface energy. The pull-off (adhesion) force could be calculated from the following equations:

1 $${F}_{ad}=-3{\rm{\pi }}{\rm{R}}\sqrt{{\gamma }_{1}{\gamma }_{2}}$$

where, *F*_ad_ is the adhesion force; *R* is the radius of the sphere; *γ*_1_ and *γ*_2_ are the surface energy of the substrate and the fine particle, respectively.

For the hydrophilic surface or at high RH, the contact behavior may be influenced significantly by the capillary bridges, which could be described by Laplace-Kelvin theoretical^57^. For various surface or particles, the capillary force shows different dependence on RH, moreover, it is relative to liquid film thickness, surface tension of the liquid and the relative vapor pressure. The capillary force for perfect sphere-on-flat geometry could be described as following:

2 $${F}_{cb}=4\pi R{\gamma }_{L}\,\cos \,\theta $$

where, *F*_cb_ is the capillary bridges, *γ*_1_ is the surface tension of the liquid, *θ* is the contact angle of liquid between the two surfaces.
